# *Hafnia alvei* Infections: Clinical Characteristics and Therapeutic Challenges of an Uncommon Pathogen

**DOI:** 10.1155/carm/8872086

**Published:** 2025-11-20

**Authors:** Jorge Eduardo Herrera Parra, Lucía Antuña Montes, Sarah Elisabeth Rodríguez Santiago, José Gutiérrez Rodríguez, Eva María López Álvarez, Natalie Burgos Bencosme

**Affiliations:** ^1^Geriatrics Department, Geriatrics Clinical Management Area, Monte Naranco Hospital, Central University Hospital of Asturias, Oviedo 33011, Spain; ^2^Health Research Institute of Asturias (ISPA), Oviedo 33011, Spain

## Abstract

*Hafnia alvei* is a rare opportunistic pathogen of the Enterobacteriaceae family, typically regarded as a gastrointestinal commensal. However, it has been implicated in serious infections, particularly in immunocompromised patients, posing diagnostic and therapeutic challenges. We present the case of a 70-year-old woman with a history of cryptogenic cirrhosis postliver transplant, complicated by sepsis and advanced pancreatic adenocarcinoma under palliative care. She developed a urinary tract infection caused by *Hafnia alvei* and *Klebsiella oxytoca*, confirmed by cultures. Despite targeted antibiotic therapy with vancomycin and ciprofloxacin, her condition deteriorated, leading to death due to oncologic disease progression. This case underscores the pathogenic potential of *Hafnia alvei*, its role in polymicrobial infections, and the importance of antimicrobial susceptibility testing. Raising awareness of this under-recognized pathogen is crucial to improving outcomes in vulnerable populations.

## 1. Introduction

*Hafnia alvei* is a Gram-negative bacterium belonging to the Enterobacteriaceae family, known as an opportunistic pathogen rarely associated with significant clinical infections in humans. Unlike more common pathogens, *H. alvei* infections have been poorly documented in the medical literature, with sporadic cases presenting both diagnostic and therapeutic challenges. This article aims to highlight a case of urinary infection by *H. alvei*, discussing its management and the clinical context in which it emerged, comparing it with other reported cases and available treatment options [1, 2].

## 2. Case Presentation

A 70-year-old woman with a history of cryptogenic cirrhosis requiring a liver transplant five years earlier, complicated by biliary anastomosis stricture and sepsis secondary to cholangitis, moderate aortic stenosis, and locally advanced unresectable pancreatic adenocarcinoma, currently in palliative care. The patient was referred to our palliative care unit due to fever without any other apparent associated symptoms. On initial assessment, she presented with poor general condition, tachycardia, and fever. Initial laboratory tests revealed leukocytosis (16,700 mm^3^) with a left shift and a procalcitonin level of 1.8 ng/mL.

Urine cultures were performed using conventional culture methods on blood agar and MacConkey agar. Identification of isolates was carried out using MALDI-TOF mass spectrometry (Bruker Biotyper). Antimicrobial susceptibility testing was performed by disk diffusion according to CLSI standards.

Cultures showed the presence of *H. alvei* and *K. oxytoca* in the urine, prompting initiation of antibiotic therapy guided by susceptibility testing with vancomycin and ciprofloxacin. Although there was initial improvement with normalization of fever and acute phase reactants, the patient died 30 days after admission due to progression of her oncological disease.

## 3. Discussion

*H. alvei* is primarily known as a commensal of the gastrointestinal tract and is rarely considered pathogenic [3, 4]. However, invasive extraintestinal infections have been reported, particularly in patients with chronic diseases or immunosuppression [5].

Documented cases are scarce but mainly involve patients with multiple comorbidities or immunosuppression, presenting with diverse clinical manifestations including gastroenteritis, bronchopneumonia, and septicemia of enteric or urinary origin [6]. A summary of previously reported cases of *H. alvei* infections, including various clinical diagnoses and affected patient populations, is presented in Table 1. Moreover, the ability of *H. alvei* to establish infections in the presence of other pathogens, as seen in our case, highlights its role in polymicrobial infections, which is an important factor to consider in the clinical management of these patients [7].

Regarding treatment, *H. alvei* has demonstrated susceptibility to aminoglycosides, third-generation cephalosporins, carbapenems, fluoroquinolones, and nitrofurantoin but shows resistance to tetracyclines, amoxicillin, ampicillin, azithromycin, and fosfomycin [8, 9]. This resistance profile underscores the importance of antimicrobial susceptibility testing to guide effective antibiotic therapy.

## 4. Conclusion

Infections caused by *H. alvei* emphasize the importance of recognizing and adequately managing opportunistic pathogens, especially in immunocompromised patients. Although generally a commensal of the gastrointestinal tract, *H. alvei* can become pathogenic under certain conditions, necessitating careful clinical management based on sensitivity testing. This analysis reinforces the need for vigilance against unusual and potentially resistant organisms to ensure better clinical outcomes for vulnerable populations.

## Figures and Tables

**Table 1:** Summary of previously reported *Hafnia alvei* infection cases.

Clinical diagnosis	Author
Urinary sepsis	Yarlagadda K.

Pyelonephritis	Cardile

Open fracture	Litrenta J.

Pneumonia	Severiche
	Méndez L.
	Begbey A.
	Lim J.
	Rodríguez E.
	Fazal B. A.
	Redondo J.

Necrotizing enterocolitis	Perinatol J.

Endocarditis	Loulergue P.
	Gallego Page J. C.

Liver transplant	Barry J. W.

Muscle abscess	Pulcini C.

Gastroenteritis	Westblom T. U.
	Reina J.
	Orden B.

Colitis	Laguna E.

Complicated cholecystitis	Guichenez P.
	Palaniswamy C.
	Hazouard E.

Hemolytic uremic syndrome	Crandall C.

Endophthalmitis	Ruiz-Moreno J. M.
	Caravalho J. Jr.

Neonatal sepsis	Casanova-Román M.

Necrotizing fasciitis	Wright W. F.
	Liliav B., Yakoub D.

Spontaneous peritonitis	Akcam F. Z.
	Fernández Peláez J. M.

## Data Availability

The data that support the findings of this study are available from the corresponding author upon reasonable request.
